# Acute anterior uveitis after topical interferon for conjunctival intraepithelial neoplasia (CIN)

**DOI:** 10.3205/oc000184

**Published:** 2021-07-20

**Authors:** Ameay V. Naravane, Jacquelyn Weber, Grishma Barucha, Joshua H. Hou

**Affiliations:** 1 University of Minnesota, Department of Ophthalmology & Visual Neurosciences, Minneapolis, United States; 2 Essentia Health Eye Care, Duluth, United States

**Keywords:** CIN, IFN alpha-2b, 5-FU, OSSN, anterior uveitis

## Abstract

**Objective:** Ocular surface squamous neoplasia (OSSN) is the most common type of non-melanocytic ocular surface tumor. Conjunctival intraepithelial neoplasia (CIN) is a type of OSSN that be medically managed by either topical interferon alpha-2b (IFN α-2b), 5-fluorouracil (5-FU), or mitomycin C. While a paradoxical response to IFN α-2b in the HIV population has been reported, we report a case of a paradoxical response in an immunocompetent individual.

**Methods:** A 65-year-old immunocompetent female presents to the clinic with CIN.

**Results:** She is started on topical IFN α-2b, resulting in an unexpected hypopyon, increased corneal epithelial defect, and increased size of the lesion. Switching to topical 5-FU resulted in decreasing size of the CIN lesion and resolution of the epithelial defect.

**Conclusions:** Topical IFN α-2b can produce a paradoxical worsening of CIN lesions in some patients. Providers should be aware of this reaction, as well as the presenting signs and symptoms, to make appropriate treatment changes when treating CIN.

## Introduction

Ocular surface squamous neoplasia (OSSN) include a broad range of neoplastic changes in the cornea and conjunctiva ranging from mild epithelial dysplasia to invasive squamous carcinoma. OSSN is the most common non-melanocytic ocular surface tumor with an overall incidence between 0.03 and 1.9 per 100,000/year in the Caucasian population and 3–3.4 per 100,000/year in the African ethnic population [1], [2]. Conjunctival intraepithelial neoplasia (CIN) is one subtype of OSSN which is characterized by neoplastic cells being confined by the basement membrane of the epithelium. CIN is estimated to occur in 0.43 cases/million/year [3].

Traditionally, surgery has been the primary treatment option for OSSN. However, recent studies have shown that there is no significant difference between surgical and medical management [4]. This has led to an increased preference of medical management among corneal specialists [5]. Most practitioners prefer topical interferon alpha-2b (IFN α-2b) as first-line medical treatment due to its low toxicity, high safety profile, and high success rate of 76–100%. 5-FU and mitomycin C are typically reserved for OSSN cases that are refractory to initial medical management [5]. Common side effects of the topical IFN α-2b drops include irritation, conjunctival hyperemia, reactive lymphoid hyperplasia, and follicular conjunctivitis [6].

Mata and colleagues described one case of an HIV-positive patient with CIN in 2014 that had a paradoxical reaction to IFN α-2b in which the CIN actually increased in size in response to medical treatment [3]. The case presented below describes another unusual reaction where there was a paradoxical growth in lesion with an associated hypopyon following topical IFN α-2b treatment in an immunocompetent individual. To our knowledge, this has not been previously reported in the literature. 

## Case description

A 65-year-old female initially presented to an outside hospital with a 6-week history of redness, pain, and tearing of her left eye. On presentation, she was diagnosed with a near total epithelial defect and suspected conjunctival intraepithelial neoplasia (CIN) affecting the limbus and peri-limbal conjunctiva for 360 degrees. The patient was started on IFN α-2b, but after 3 weeks, there was progressive growth of the lesion with extension of the neoplasm temporally. The patient was then referred to our institution for further evaluation and treatment. Past ocular history was positive for myopia of both eyes and a left eyelid injury following a dog bite as a child. The patient’s past medical history was positive for well-controlled hypertension. She had a 40-pack-year smoking history and admitted to occasional alcohol use, though she denied any intravenous or recreational drug use. She was HIV-negative and had no pertinent family history of malignancy.

**Figure 1:**
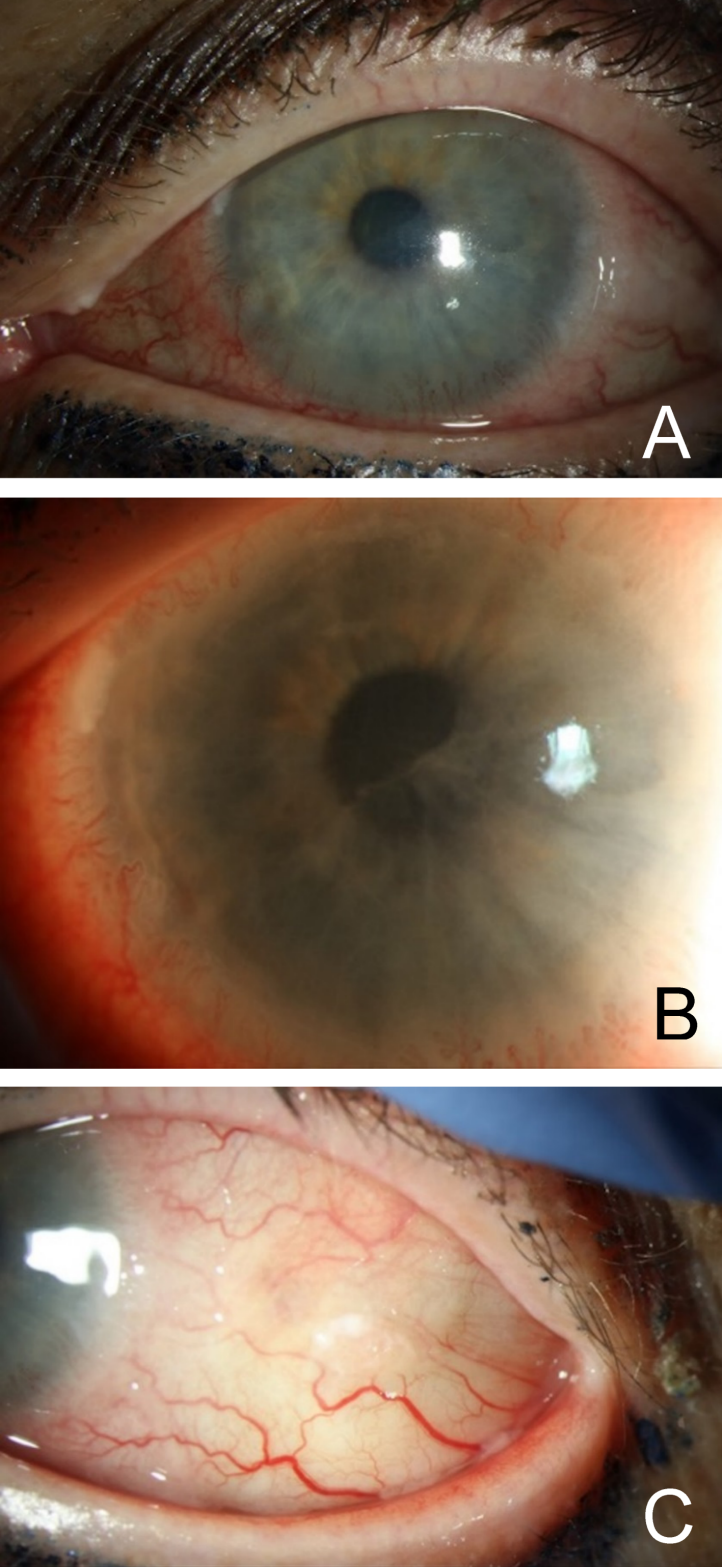
A) CIN with 360-degree limbal involvement; B) Perilimbal gelatinous lesion with vascular loops; C) CIN with perilimbal lesion extending to the temporal bulbar conjunctiva

On initial presentation to our clinic, the patient had a large inferior corneal epithelial defect (6x3.5 mm) with mild subepithelial haze in her left eye. There was 360 degrees of gelatinous limbal thickening with vascular loops suspicious for CIN (Figure 1A, B (Fig. 1)). Also noted was significant extension of the lesion temporally onto the lateral bulbar conjunctiva (Figure 1C (Fig. 1)). Due to the 360 degrees of limbal involvement and risk of severe limbal stem cell deficiency with surgical excision of the lesion, the patient was continued on the IFN α-2b drops (1 million IU/ml) four times a day. A bandage contact lens was placed to promote re-epithelialization of the corneal epithelial defect. Topical ofloxacin was added twice daily to the left eye for the epithelial defect.

**Figure 2:**
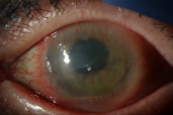
Enlarging epithelial defect and hypopyon

A month after the initial presentation, the patient complained of increasing left eye pain and redness and returned urgently to the clinic. Slit lamp examination showed a new hypopyon, engorged iris vessels, and posterior synechiae consistent with acute non-granulomatous anterior uveitis (Figure 2 (Fig. 2)). No keratic precipitates or corneal infiltrates were noted. An infectious and autoimmune workup was performed. All bloodwork, including quantiferon gold, RPR, and HLA-B27, was negative. CRP was slightly elevated at 0.5 (normal range 0.0–0.3). There was also increased thickening of the CIN and notable extension of the lesion 360 degrees onto the cornea. There was no posterior involvement on B-scan ultrasound. In the setting of a new hypopyon, IFN α-2b drops were held, and the bandage contact lens was removed. The patient was started on prednisolone 1% ophthalmic drops four times a day and continued on topical ofloxacin four times a day. The following day, the hypopyon had significantly improved; however, the CIN continued to progress with worsening of the corneal epithelial defect (6.5x4 mm). A Prokera amniotic membrane was placed on the left eye.

Two weeks later, the patient described less pain and less photophobia. The hypopyon was resolved, but the patient continued to have a larger persistent epithelial defect. The previous Prokera amniotic membrane had dissolved. The patient then received a repeat Prokera amniotic membrane and temporary suture tarsorrhaphy. Prednisolone 1% was also decreased to three times daily to promote re-epithelialization. IFN α-2b drops continued to be held.

**Figure 3:**
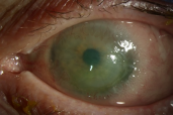
Enlargement of CIN, increased corneal epithelial defect size

The patient was seen in follow-up three weeks later, and significant growth of the CIN was noted (Figure 3 (Fig. 3)). Given the uveitic reaction to IFN α-2b, the patient was switched to 5-FU drops four times per day in her left eye. Prednisolone 1% drops were tapered to twice daily. 

**Figure 4:**
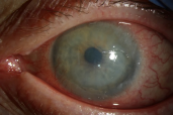
Diminished CIN two weeks after starting 5-FU; note regressing superior vessels

Two weeks later, there was interval improvement of the CIN, and 5-FU drops four times daily was continued. The epithelial defect remained stable at 5.5x6 mm (Figure 4 (Fig. 4)).

**Figure 5:**
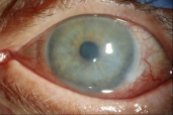
Diminished CIN, resolving epithelial defect 1.5 months after 5-FU therapy initiation

One month later, the epithelium began to heal, albeit slowly, as the CIN continued to improve. Prednisolone 1% drops were tapered to once daily. Ofloxacin was continued twice daily. An Ambiodisk amniotic membrane and Kontur lens were placed in the left eye for the persistent epithelial defect. After six weeks of topical 5-FU, the patient was noted to have near resolution of the CIN (Figure 5 (Fig. 5)). She was then started on oral cimetidine 400 mg twice daily for its immunomodulatory effects [6].

**Figure 6:**
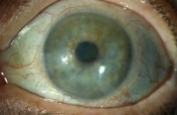
Resolution of CIN, small inferior corneal neovascularization, resolved epithelial defect with scarring and some residual amniotic membrane inferotemporally

Finally, six months after initial presentation, the persistent corneal epithelial defect was resolved after suture tarsorrhaphy, multiple amniotic membranes, and bandage contact lenses (Figure 6 (Fig. 6)). The patient completed a two-month course of oral cimetidine (400 mg twice daily dosing). To date, there has been no recurrence of CIN.

## Discussion

To the authors’ knowledge, this is the first case reported in the literature of an acute anterior uveitis in the setting of topical IFN α-2b therapy for CIN. Temporary cessation of the medication and initiation of topical prednisolone resulted in rapid resolution of the uveitis. However, rapid progression of the CIN was noted afterwards. It is unclear whether the addition of topical steroids or just the cessation of IFN α-2b was responsible for the rapid growth of the CIN. Prior to onset of the uveitis, the lesion appeared refractory to the topical IFN. However, the rapid growth after cessation of the drug may indicate that the medication was at least limiting the growth of the lesion. When the patient was switched to 5-FU, the CIN regressed completely. This patient fortunately did not suffer any permanent visual loss from the acute anterior uveitis or interval progression of CIN; however, it may have led to visual loss if not for the reaction being properly identified and treated, and for the patient being promptly switched to an alternate therapy.

There was a low suspicion for ocular sarcoidosis based on unilateral disease. However, a chest X-ray, angiotensin-converting enzyme (ACE) level, and lysozyme were not obtained.

The cause of the patient’s reaction to the topical IFN α-2b is not known. Interferons have been shown in the literature to reduce viral replication and inhibit cancer cell mitosis. Though topical IFN α-2b is an established therapy for CIN, our patient was unresponsive to the IFN treatment prior to developing uveitis. Our patient subsequently had a good response to topical 5-FU. 5-FU works through a different mechanism than IFN by specifically inhibiting thymidine synthase, thus directly preventing DNA and RNA replication in cells [5]. It is unclear if the anterior uveitis that was observed was a direct immune reaction to the IFN α-2b itself or an immune response to the refractory CIN (or associated viral shedding).

Although CIN has not been reported to be associated with uveitis, CIN has been associated with human papillomavirus (HPV) infection. Multiple studies have implicated HPV as a pathogenic co-factor in addition to UV and HIV co-infection in the pathogenesis of conjunctival intraepithelial neoplasia [7]. HPV, in turn, has been associated with Behçet’s disease, a systemic inflammatory disorder characterized by oral and genital sores, arthritis, and uveitis [8]. Furthermore, at least 24 cases of bilateral uveitis have been reported following administration of quadrivalent HPV vaccine, which is prepared from the highly purified virus-like particles of the major capsid (L1) protein of HPV types 6, 11, 16, and 18 [9]. It is possible that shedding of viral proteins following treatment of CIN with topical IFN α-2b led to the observed acute anterior uveitis in our patient. However, a direct immune reaction to the medication also remains a possibility.

In our patient, the CIN was very extensive on presentation. Her persistent epithelial defect further suggested some pre-existing degree of limbal dysfunction stemming from the extensive limbal involvement of her CIN. Surgical excision would have likely induced significant limbal stem cell deficiency, causing further complications such as corneal scarring and loss of vision. As a result, topical IFN α-2b was used as the primary treatment. The main benefit of this approach was that the patient’s limbus was able to fully recover after successful treatment with 5-FU. The downside of this approach was that no histopathological analysis of the neoplasia was possible. Therefore, the diagnosis of CIN was not confirmed by biopsy or histopathology. This also precluded HPV testing of the lesion. As a result, our ability to draw conclusions about the role of HPV in this case is limited.

In summary, topical IFN α-2b therapy for CIN may cause an acute anterior non-granulomatous uveitis in immunocompetent patients. Identification of this reaction is crucial to proper treatment and management including switching to alternate topical therapy (such as 5-FU).

## Notes

### Competing interests

The authors declare that they have no competing interests.
